# The predictive value of TyG index in patients with vertebrobasilar system thrombectomy

**DOI:** 10.3389/fneur.2025.1597323

**Published:** 2025-08-15

**Authors:** Jiazhen Wang, Honggang Ma, Xuanfei Jiang, Ru Pan, Bing Zhang, Ying Liu

**Affiliations:** ^1^Department of Neurology, Huzhou Central Hospital, The Fifth School of Clinical Medicine of Zhejiang Chinese Medical University, Huzhou, China; ^2^Department of Pathology, Huzhou Central Hospital, The Fifth School of Clinical Medicine of Zhejiang Chinese Medical University, Huzhou, China

**Keywords:** triglyceride-glucose index, vertebrobasilar system thrombectomy, modified Rankin score, ischemic stroke, metabolic-nutritional status

## Abstract

**Introduction:**

The triglyceride-glucose (TyG) index, a robust surrogate marker of metabolic dysregulation reflecting both insulin resistance and lipid-glucose homeostasis, has emerged as a significant predictor of cerebrovascular outcomes. Given the critical role of metabolic-nutritional status in post-stroke recovery, we supposed that the TyG index may predict the prognosis of ischemic stroke patients who underwent thrombectomy in the posterior circulation.

**Methods:**

We studied 60 patients with cerebral infarction who underwent emergency posterior circulation interventional thrombectomy at a comprehensive stroke center from January 2018 to July 2024. The TyG index was used as the cut-off value of 8.53, and the formula was calculated as TyG index = ln [fasting glucose(mg/dL) × fasting triglycerides(mg/dL) /2]. Univariate analysis and multivariate logistic regression were used to adjust for age, the National Institutes of Health Stroke Scale (NIHSS) score at onset, APOA-1, and diabetes. A modified Rankin scale score of 0–2 at 90 days defined a good functional outcome, and the incidence of death within 90 days was investigated.

**Results:**

The number of patients with good functional outcome in the high TyG index group was significantly less than that in the low TyG index group (adjusted OR 6.85, 95%CI 1.83, 32.13, *p* = 0.008). TyG index was significantly associated with 90-day mortality (adjusted OR: 5.113, 95%CI 1.274 to 20.519, *p* = 0.021).

**Discussion:**

This study found that TyG index was linearly correlated with the 90-day neurological recovery in patients with acute posterior circulation cerebral infarction after interventional thrombectomy. The higher the TyG index, the worse the neurological recovery and the higher the risk of death.

## Introduction

Posterior circulation stroke, characterized by cerebral infarction in the vertebrobasilar system, and is collectively referred to as vertebrobasilar artery occlusion (VBAO), with basilar artery occlusion (BAO) being a common form of this condition (1), presents distinct metabolic challenges due to the high energy demands of brainstem and cerebellar structures. Although posterior circulation strokes account for a relatively small proportion of all cerebral infarctions, they often lead to severe neurological deficits due to the involvement of critical structures such as the brainstem, cerebellum, and occipital lobes. In severe cases, these strokes can be life-threatening (2). Research has shown that among patients with acute BAO who underwent interventional thrombectomy, half still did not achieve a favorable outcome within 90 days, and approximately 40% progressed to a fatal outcome. Notably, patients with poor collateral circulation faced worse clinical prognoses (3, 4). Despite advances in imaging techniques, including computed tomography (CT) and magnetic resonance imaging (MRI), the diagnosis of posterior circulation cerebral infarction has significantly improved; however, these methods do not predict functional recovery. Therefore, there is an urgent need for sensitive and reliable prognostic indicators to guide clinical treatment (5).

Crucially, emerging evidence suggests that metabolic-nutritional status may determine post-stroke recovery through mitochondrial bioenergetics, oxidative stress regulation, and neurovascular unit maintenance (6). However, current prognostic models predominantly focus on anatomical parameters, neglecting the critical dimension of systemic metabolism. The triglyceride-glucose (TyG) index, calculated as ln[fasting triglycerides (mg/dL) × fasting glucose (mg/dL) /2], serves as a composite biomarker of metabolic flexibility by integrating lipid handling efficiency and glucose homeostasis (5). Beyond quantifying insulin resistance (7), an elevated TyG index reflects fundamental metabolic derangements, including: (1) impaired adipose tissue lipolysis regulation, (2) ectopic lipid deposition, and (3) neuroendocrine dysregulation of nutrient partitioning (8). These disturbances may exacerbate the cerebral energy crisis during stroke recovery by limiting substrate availability for neuronal repolarization and axonal regeneration (9). As research progresses, the TyG index has been shown to be associated with various metabolic abnormalities and cardiovascular diseases (CVD). Studies by Wang et al. (10) and Zhu et al. (11)have demonstrated that the TyG index can significantly predict the likelihood of severe cardiovascular and cerebrovascular events in patients with acute coronary syndrome (ACS) when incorporated into a mature and reliable risk assessment model. In recent years, increasing attention has been given to the relationship between the TyG index and cerebrovascular diseases, particularly its prognostic value in cerebral infarction (12). For example, Hou et al. (13) found that all-cause mortality and risk of stroke recurrence were significantly increased in patients with ischemic stroke with higher TyG index through the analysis of data from CNSR II. In addition, Nam et al. (14) noted that patients with higher TyG indices were more likely to experience early neurological deterioration following acute ischemic stroke. A large-scale meta-analysis revealed that, among patients with anterior circulation cerebral infarction, those with a high TyG index had significantly worse outcomes, including higher mortality rates and an increased likelihood of recurrent cerebral infarction (15). For patients with cerebral infarction resulting from large-vessel occlusion in the posterior circulation, emergency interventional thrombectomy remains the most effective treatment due to the complexity of vascular anatomy and the relatively low incidence rate (16). However, the predictive value of the TyG index for functional recovery following thrombectomy remains unclear.

Based on the above-mentioned research, we assume that the TyG index may predict the prognosis of AIS patients who underwent thrombectomy in the posterior circulation by quantifying their blood glucose and lipid levels. To test this hypothesis, we conducted a cohort study of 60 VBAO patients undergoing thrombectomy (2018–2024), stratified by a TyG index threshold of 8.53. Using multivariable models adjusted for APOA-1 (a key regulator of reverse cholesterol transport) and diabetes status, we specifically examined the relationship between the TyG levels and 90-day functional outcomes, and mortality risk.

## Methods and materials

### Study population

This is a retrospective observational cohort study from a single center, and the prediction value of the TyG index for the prognosis of the vertebrobasilar artery system is assessed. Sixty acute ischemic stroke (AIS) patients who underwent emergency vertebrobasilar system thrombectomy from January 2018 to July 2024 were enrolled. Exclusion criteria were as follows: (1) previous modified Rankin scale score ≥2; (2) a lack of follow-up information on outcomes; (3) progressive cerebral infarction; (4) failure of vascular recanalization (17). A total of sixty-seven patients underwent posterior circulation thrombectomy in our center during the study period. Five patients were excluded because of progressive cerebral infarction, and two patients were excluded because of missing follow-up data. The research samples were selected based on strict diagnostic criteria. For instance, only patients with definite posterior - circulation cerebral infarction, a clear onset time, and successful recanalization after thrombectomy were included. This approach, to a certain extent, ensured the pertinence and homogeneity of the samples. Although the sample size was relatively smaller compared to some large - scale studies, in the context of researching the rare disease of large-vessel occlusion in the posterior circulation and predicting subsequent functional recovery, the samples are fairly representative.

All participants received acute stroke management based on international and domestic guidelines. In each case, the angioneurologist was responsible for determining whether thrombolytic therapy should be carried out. In the process of internal treatment, the interventional physician chose the intervention strategy for each patient.

### Data collection and clinical variables

Baseline demographic, clinical, imaging, and laboratory data were collected for all enrolled patients. Baseline data included: (1) demographic data, such as age and gender; (2) Past medical histories, including previous stroke, hypertension, diabetes mellitus, current smoker status, atrial fibrillation, and use of anticoagulants; (3) Stroke characteristics included the NIHSS score, stroke modified Rankin scale (mRS) score, posterior circulation Alberta Stroke Program Early CT score (PC-ASPECTS), Basilar Artery Treatment and Management (BATMAN) score, stroke classification and occlusion location; (4) Reperfusion therapy, including thrombolytic therapy or not, time from onset to thrombolysis, time from onset to puncture, time from puncture to recanalization, number of thrombectomy attempts and thrombectomy methods; (5) Laboratory data, including fasting lipoprotein (a), fasting apolipoprotein A-1 (APOA-1), fasting apolipoprotein B(APOB), APOA-1/APOB, fasting low-density lipoprotein, fasting blood glucose, and fasting triglyceride. We collected a relatively large number of different lipid indices in the baseline table. The reason is that some lipid indices are independent risk factors for cerebral infarction (18), and this not only allows us to observe their differences in the groups but also can be used for subsequent multivariate regression analysis.

The TyG index was calculated in the following way. During the first 24 h after the onset of illness, each patient fasted for more than 8 h overnight, and blood glucose and lipid levels were measured in the early morning hours. The TyG index was calculated: TyG index = ln[fasting glucose(mg/dL) × fasting triglycerides(mg/dL) /2] (19). Characteristics of patients with ‘Low TyG’ and ‘High TyG’ indices were then compared using≥8.53 as a cut-off value.

### Clinical outcomes

We evaluated the primary clinical outcomes of favorable functional outcome status defined by the modified Rankin Scale (mRS) score of 0–2 (20, 21). Secondary outcomes included mRS scores 0–1, 0–3 and 0–4 at 90 days. Safety outcomes included death, symptomatic intracranial hemorrhage (sICH), procedural complications, and severe adverse events within 90 days.

### Statistical analysis

The analysis included measures such as mean (standard deviation) for normally distributed variables and median (interquartile range, IQR) for non-normally distributed variables for continuous variables, and frequencies and percentages for categorical variables. Normality tests and Quantile-Quantile (QQ) plots were used to assess the distribution of the data, and appropriate descriptive statistics methods were applied to both normally and non-normally distributed variables. Group comparisons for continuous variables with normal distribution were performed using Welch’s t-test or ANOVA, and non-normally distributed variables were compared using the Wilcoxon rank-sum test or Kruskal-Wallis test. For comparison between groups of categorical data, we used the Fisher exact test for expected frequencies <5; otherwise, we used the Chi-squared test. We employed rigorous statistical methods to analyze the limited data, aiming to unearth the latent relationships and differences within the data, thus enhancing the credibility of the results.

In our study, all statistical analyses were performed using the R software (version 4.2.2). Variables were screened by Elastic net regression. Univariate Logistic regression analysis was performed to assess the association between each individual factor and functional outcome. Multivariate Logistic regression analysis was performed to determine the independent factors significantly associated with mRS scores, while adjusting for potential confounders. All variables from the univariate analysis were included in the multivariate Logistic regression model, regardless of their significance in the univariate analysis. This comprehensive approach ensured that all potential influencing factors were considered and adjusted for, providing a more accurate estimation of the independent clinical factors that significantly impacted mRS scores.

## Result

### Study participants and baseline characteristics

A total of 60 patients satisfying the eligibility criteria were included in the final analysis. The baseline characteristics of the study population were summarized (Table 1). The baseline characteristics of patients, categorized by clinical outcomes, reveal several key findings. The median age was slightly higher among those with poor clinical outcomes (73 years) compared to those with good outcomes (70 years), though this difference was not statistically significant (*p* = 0.099). Gender distribution was similar between groups with a predominance of males in both (73.9% in good outcomes, 70.3% in poor outcomes). Notably, a significant difference was observed in Apolipoprotein A-1 levels, with better outcomes associated with higher levels (1.26 ± 0.17 vs. 1.14 ± 0.17, *p* = 0.015). Fasting glucose levels presented a significant disparity, showing lower levels in good outcomes (5.5) compared to the poor (7.6, *p* < 0.001). Additionally, the TyG index was more favorable in patients with good outcomes, with a significant portion having an index below 8.53 (69.6% vs. 37.8%, *p* = 0.017). The baseline NIHSS score showed a modest significance, with median values clustering around 36 for both groups, but with variation in distribution (*p* = 0.045). Other variables, such as history of hypertension, diabetes, smoking, drinking, ischemic stroke, and thrombolysis interventions, presented no significant differences between the groups, suggesting similar exposure profiles. No significant differences were observed in triglycerides, low-density lipoprotein, and lipoprotein a levels.

**Table 1:** Patient demographics and baseline characteristics.

Characteristic	mRS scores		*p*-value
	0–2, *N* = 23^1^	3–6, *N* = 37^1^	
Age	70 (64, 75)	73 (68, 79)	0.099^2^
Sex			0.761^3^
Female	6 (26.1%)	11 (29.7%)	
Male	17 (73.9%)	26 (70.3%)	
Smoking			0.597^3^
No	14 (60.9%)	25 (67.6%)	
Yes	9 (39.1%)	12 (32.4%)	
Drinking			0.603^3^
No	16 (69.6%)	28 (75.7%)	
Yes	7 (30.4%)	9 (24.3%)	
History of hypertension			>0.999^4^
No	4 (17.4%)	6 (16.2%)	
Yes	19 (82.6%)	31 (83.8%)	
History of atrial fibrillation			0.749^4^
No	19 (82.6%)	28 (75.7%)	
Yes	4 (17.4%)	9 (24.3%)	
History of anticoagulants			>0.999^4^
No	21 (91.3%)	34 (91.9%)	
Yes	2 (8.7%)	3 (8.1%)	
History of diabetes mellitus			0.090^3^
No	18 (78.3%)	21 (56.8%)	
Yes	5 (21.7%)	16 (43.2%)	
History of ischemic stroke			>0.999^4^
No	20 (87.0%)	33 (89.2%)	
Yes	3 (13.0%)	4 (10.8%)	
Lipoprotein a	16 (12, 29)	12 (7, 28)	0.210^2^
Apolipoprotein A-1	1.26 ± 0.17	1.14 ± 0.17	0.015^5^
Apolipoprotein B	0.85 (0.67, 0.95)	0.74 (0.58, 0.91)	0.221^2^
Low density lipoprotein	76 (56, 105)	74 (59, 107)	0.964^6^
Triglycerides	1.05 (0.78, 1.52)	1.15 (0.68, 1.57)	>0.999^2^
Fasting glucose	5.5 (4.8, 6.0)	7.6 (6.0, 8.9)	<0.001^2^
APOA-1/APOB	1.44 (1.20, 1.83)	1.45 (1.26, 1.88)	0.885^2^
Cause of stroke			0.849^4^
Atherosclerosis	12 (52.2%)	18 (48.6%)	
Cardiac embolism	7 (30.4%)	13 (35.1%)	
Other causes	3 (13.0%)	3 (8.1%)	
Unknown	1 (4.3%)	3 (8.1%)	
Time from symptom onset to groin puncture	290 (198, 419)	280 (206, 415)	0.867^2^
Time from symptom onset to recanalization	355 (260, 483)	352 (266, 498)	0.773^2^
Puncture to recanalization time	60 (46, 78)	66 (38, 89)	0.808^2^
Door to recanalization time	177 (150, 214)	178 (152, 270)	0.727^2^
First thrombectomy attempt			0.593^4^
Aspiration	4 (17.4%)	9 (24.3%)	
Stenting	12 (52.2%)	21 (56.8%)	
Stenting+stenting	7 (30.4%)	7 (18.9%)	
Location of intracranial artery occlusion			0.876^4^
Distal	1 (4.3%)	2 (5.4%)	
Middle	4 (17.4%)	5 (13.5%)	
Tip of the basilar artery occlusion	18 (78.3%)	30 (81.1%)	
Number of thrombectomy maneuvers per-intervention			0.580^3^
1	12 (52.2%)	22 (59.5%)	
2	11 (47.8%)	15 (40.5%)	
Triglyceride-glucose index			0.017^3^
<8.53	16 (69.6%)	14 (37.8%)	
≥8.53	7 (30.4%)	23 (62.2%)	
Intravenous thrombolysis			0.500^3^
No	11 (47.8%)	21 (56.8%)	
Yes	12 (52.2%)	16 (43.2%)	
Tirofiban			0.682^3^
No	15 (65.2%)	26 (70.3%)	
Yes	8 (34.8%)	11 (29.7%)	
BATMAN score			0.524^3^
0–8	15 (65.2%)	27 (73.0%)	
9–10	8 (34.8%)	10 (27.0%)	
PC-ASPECTS			0.391^3^
0–8	4 (17.4%)	10 (27.0%)	
9–10	19 (82.6%)	27 (73.0%)	
Baseline NIHSS score	36.0 (25.5, 36.0)	36.0 (36.0, 36.0)	0.045^2^

^1^Median (interquartile range, IQR); *n* (%); Mean ± SD.

^2^Wilcoxon rank sum test.

^3^Pearson's Chi-squared test.

^4^Fisher's exact test.

^5^Welch two sample *t*-test.

^6^Wilcoxon rank sum exact test.

Regarding reperfusion therapy, all patients underwent endovascular thrombectomy, with more than half of the patients receiving stent thrombectomy (*n* = 33, 55%), while the remaining patients underwent aspiration techniques (*n* = 13, 21.7%) and a combination of aspiration and stent thrombectomy (*n* = 14, 23.3%). Approximately half of the patients received intravenous thrombolysis (IVT) (*n* = 28, 46.7%, *p* = 0.5), and a minority received tirofiban antiplatelet therapy (*n* = 19, 31.7%, *p* = 0.682). These treatments did not significantly affect the prognosis of the patients after three months. The average time from symptom onset to puncture was 287.5 min (IQR: 205.75–413, *p* = 0.752), the average time from symptom onset to recanalization was 353.5 min (IQR: 262–490, *p* = 0.672), the average time from puncture to recanalization was 65.5 min (IQR: 40–79, *p* = 0.454), and the average time from hospital arrival to recanalization was 177.5 min (IQR: 149.75–220, *p* = 0.471).

### Functional outcomes by the TyG index

For 90 - day clinical outcomes, compared with patients with high TyG index, patients with low TyG index had mRS scores of 0–1 (33.3% vs. 10%, *p* = 0.028), 0–3 (70% vs. 26.7%, *p* = 0.017). *p* < 0.001 and 0–4 (73.3% vs. 46.7%, *p* = 0.038). After adjusting for age, NIHSS score at onset, APOA-1 and diabetes, TyG index remained an independent predictor of good outcome by multivariate logistic regression (Table 2).

**Table 2:** Comparison of clinical outcomes and safety between low ‘low TyG’ and ‘high TyG’ patients.

Characteristic	OR^1^	95% CI^1^	*p* value	adjusted OR^1^	95% CI^1^	*P* value
Clinical outcomes						
mRS scores at 90 days according, range — no. (%)						
0 or 1	4.5	1.09, 18.50	0.037	7.459	1.43, 38.87	0.017
0 to 3	6.42	2.08, 19.76	0.001	16.572	3.34, 82.14	0.001
0 to 4	3.14	1.07, 9.27	0.038	5.319	1.37, 20.70	0.016
Imaging outcomes						
sICH at 24–72 h no. (%)	1.18	0.38, 3.63	0.775	0.958	0.24, 3.76	0.951
Safety outcomes						
Death within 90 days — no. (%)	3.06	0.97, 9.66	0.057	5.113	1.27, 20.52	0.021
sICH at 24–72 h — no. (%)	1.39	0.28, 6.70	0.688	1.334	0.27, 6.70	0.727
Severe adverse events	1.46	0.44, 4.86	0.543	1.22	0.32, 4.60	0.769

^1^OR, odds ratio; CI, confidence interval.

At the same time, patients with high TyG had a higher risk of death within 90 days. Using multivariate logistic regression, after adjusting for age, NIHSS score at onset, APOA-1, and diabetes, TyG was also found to be an independent predictor of mortality risk (adjusted OR 5.113, 95% CI 1.274 to 20.519, *p* = 0.021). Compared with the high Tyg group, patients in the low Tyg group had a lower risk of ICH within 90 days, a lower incidence of symptomatic intracranial hemorrhage (sICH) at 24–72 h, and a lower number of adverse events. However, these differences were not statistically significant (Table 2).

**Figure 1:**
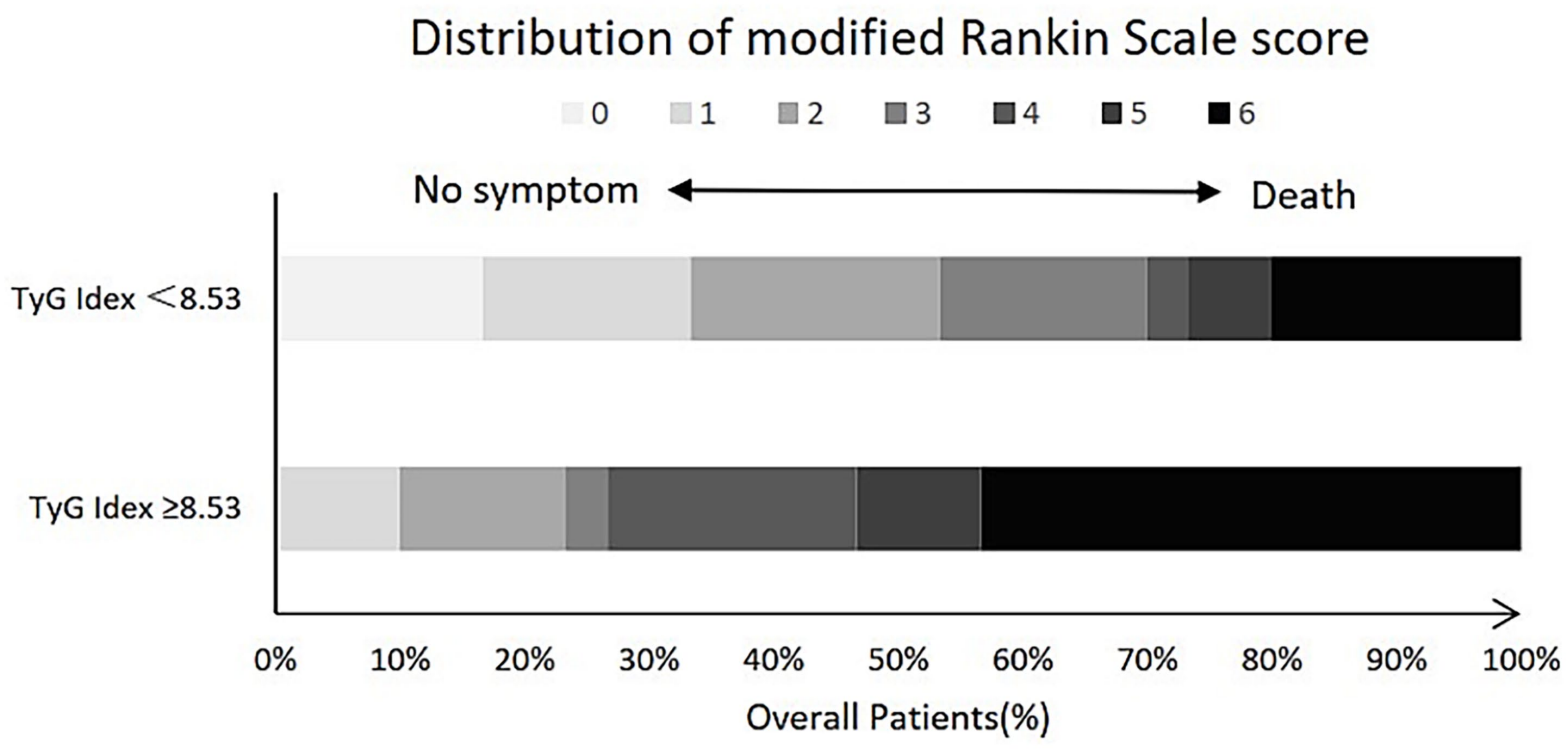
Comparison of mRS distribution between “low TyG” and “high TyG” patients.

We also explored the overall distribution of mRS scores at 90 days in the high and low TyG index groups (Figure 1).

### Logistic regression analyses

**Figure 2:**
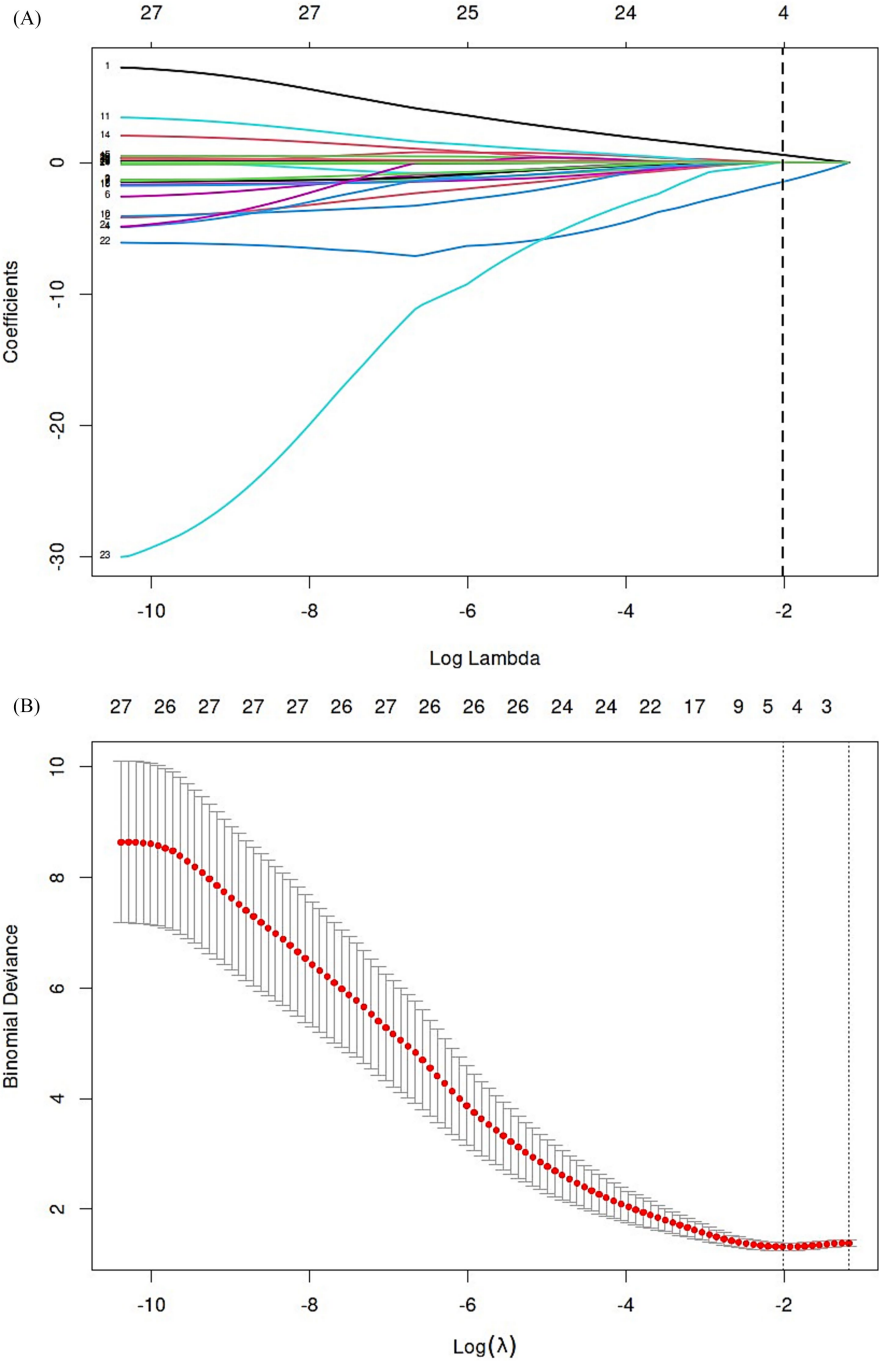
Elastic net regression and tenfold cross-validation were used to select the radiomics features. **(A)** Elastic net coefficient profiles of the radiomic features. **(B)** Optimal feature selection of mRS.

**Figure 3:**
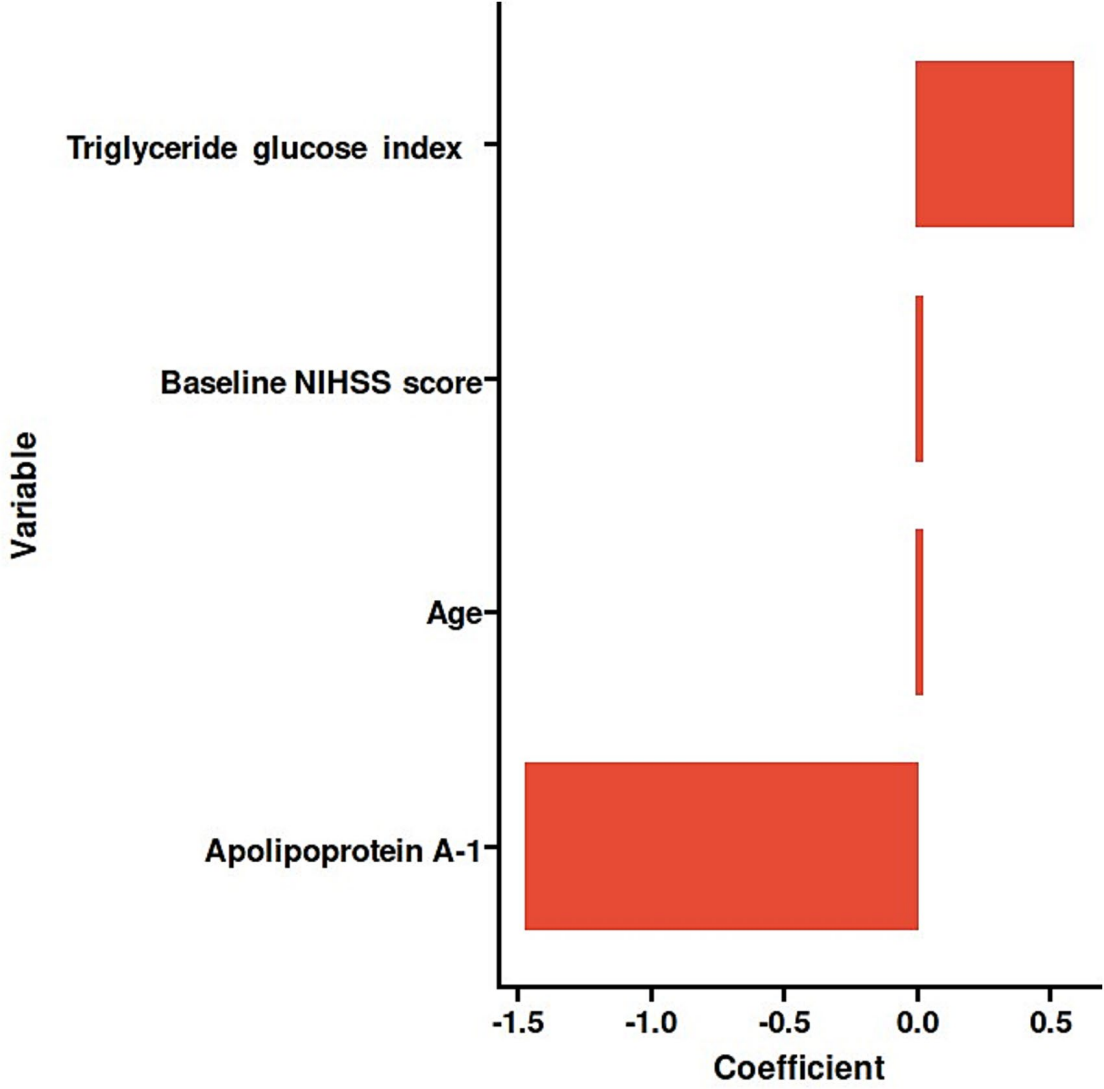
Histogram of the coefficients of the selected features.

The 27 variables were screened by Elastic net regression (Figure 2A). When *λ* = 0.134, the following variables were selected for multivariate analysis: TyG index (Coefficient = 0.58221679), Age (Coefficient = 0.01415229), Apolipoprotein A-1 (Coefficient = −1.46789936), Baseline NIHSS score (Coefficient = 0.01621730) (Figure 2B). The details of the selected features were presented (Figure 3).

**Figure 4:**
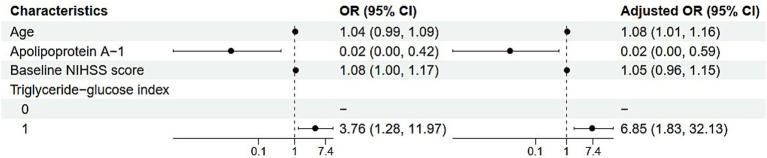
Forest plot of univariate and multivariate analysis of influencing factors.

In the analysis of factors associated with the outcome measured by mRS scores, several key findings emerged (Table 3). Each one-year increase in age was associated with an 8% increase in the odds of the outcome (adjusted OR, 1.08; 95% CI, 1.01–1.16; *p* = 0.027), indicating a significant association. The level of APOA-1 demonstrated a protective effect, where each unit increase was associated with a 98% reduction in odds (adjusted OR, 0.02; 95% CI, 0.00–0.59; *p* = 0.034). In contrast, while the baseline NIHSS score appeared to suggest a 5% increase in odds per unit increase, this association was not statistically significant (adjusted OR, 1.05; 95% CI, 0.96–1.15; *p* = 0.291). Regarding the TyG index, individuals with a value of ≥8.53 had significantly higher odds of the outcome compared to the reference group, with an adjusted odds ratio of 6.85 (95% CI, 1.83–32.13; *p* = 0.008), highlighting a strong and statistically significant relationship (Figure 4).

**Table 3:** Univariate and multivariate analysis of influencing factors (Logistic regression).

Characteristic	Univariable			Multivariable		
	OR^1^	95% CI^1^	*p*-value	OR^1^	95% CI^1^	*P* value
Age	1.04	0.99, 1.09	0.115	1.08	1.01, 1.16	0.027
Apolipoprotein A-1	0.02	0.00, 0.42	0.019	0.02	0.00, 0.59	0.034
Baseline NIHSS score	1.08	1.00, 1.17	0.051	1.05	0.96, 1.15	0.291
TyG index						
<8.53	—	—		—	—	
≥8.53	3.76	1.28, 11.97	0.019	6.85	1.83, 32.13	0.008

^1^OR, odds ratio; CI, confidence interval.

Null deviance = 79.9; Null df = 59.0; Log-likelihood = −30.2; AIC = 70.3; BIC = 80.8; Deviance = 60.3; Residual df = 55; No. Obs. = 60.

## Discussion

This real-world study investigated the predictive value of the TyG index for postoperative functional recovery in patients with posterior circulation ischemic stroke undergoing thrombectomy. The results indicate that a low TyG index is significantly associated with better functional recovery and reduced mortality in these patients, supporting our hypothesis. Compared to the high TyG index group, the low TyG index group demonstrates an 8- to 16-fold reduction in the risk of poor functional outcomes. Furthermore, no significant correlation was observed between the TyG index sICH outcomes within 36 h. These findings provide preliminary evidence supporting the predictive value of the TyG index for postoperative neurological recovery in patients with VBAO. Despite the limited sample size, this study offers valuable insights and serves as a foundation for future research in this area.

The TyG index, a composite indicator combining fasting blood glucose and triglycerides, serves as a marker of insulin resistance and is classified as an insulin-related metric (22). In recent years, increasing attention has been given to the relationship between insulin-related indicators, including the TyG index, and ischemic stroke, as glucose and lipid metabolism abnormalities exacerbate cardiovascular and cerebrovascular atherosclerosis. Insulin measurement is not routine in clinical practice, and various methods exist for its assessment, such as the hyperinsulinemic euglycemic clamp (HEC), the minimal model of the intravenous glucose tolerance test (IVGTT), the homeostasis model assessment (HOMA), and the quantitative insulin sensitivity check index (QUICKI). These methods are all based on the interaction between the glucose-insulin system (23, 24). Among them, HEC is considered the “gold standard” for measuring insulin resistance because it requires minimal additional parameters to determine blood glucose levels (25). However, accurate HEC results depend on highly skilled operators and specialized testing facilities (26, 27). In contrast, the TyG index is a practical alternative that can be routinely measured in clinical practice, demonstrating excellent consistency across different platforms (28).

The association between a low TyG index and a reduced risk of poor functional outcomes may be attributed to several factors. The TyG index, calculated using fasting blood glucose and triglycerides, incorporates two components independently linked to stroke risk (9). In insulin resistance states, microglial cell function is disrupted, causing polarization toward the pro-inflammatory M1 phenotype and the release of inflammatory factors such as TNF-*α* (Tumor Necrosis Factor-alpha) and IL-1β (Interleukin-1 beta), which result in cellular damage. Concurrently, an increase in neutrophil levels damages the blood–brain barrier, contributing to worse stroke outcomes (29, 30). Moreover, insulin resistance accelerates atherosclerosis through various mechanisms, including promoting vascular smooth muscle cell proliferation and migration, enhancing lipid deposition, narrowing or occluding blood vessels, and impairing cerebral blood flow, all of which negatively affect patient prognosis (31, 32). Research also indicates that platelets exhibit hyperactivity under IR conditions, potentially forming pathological clots that reduce the efficacy of reperfusion therapy (33, 34). Additionally, elevated triglyceride levels are recognized as a stroke risk factor, as triglyceride-rich lipoproteins (TRLs) exacerbate atherosclerosis through their toxic and pro-inflammatory effects (22). The lipolytic products of TRLs can induce endothelial cell apoptosis and accelerate atherosclerosis progression by increasing oxidative stress via activation of the mitogen-activated protein kinase (MAPK) signaling pathway, ultimately heightening stroke risk and hindering recovery outcomes (35).

Our findings revealed an association between the TyG index and the prognosis of posterior circulation AIS patients following thrombectomy. A prospective multicenter study involving 914 patients demonstrated a strong correlation between the TyG index and the functional outcomes of AIS patients treated with IVT. Patients with a higher TyG index exhibited worse functional outcomes, while no significant differences were observed in the incidence of sICH (36). Similarly, Minwoo Lee’s team confirmed these findings through a retrospective study, showing that a high TyG index was independently associated with poor functional outcomes three months after ischemic reperfusion therapy in AIS patients and was linked to an increased frequency of early neurological deterioration (37). Another study further established that the TyG index serves as an independent factor influencing early neurological recovery after thrombolytic therapy in AIS patients (38). Consistent results were reported in a Chinese study: utilizing data from CNSR II, a prospective cohort of 16,310 ischemic stroke patients, multivariable Cox regression and logistic regression analyses indicated that the TyG index was associated with a higher risk of neurological deterioration and increased all-cause mortality in stroke patients (39). Additionally, studies in Chinese AIS patients identified a higher TyG index as an independent predictor of mortality within three and twelve months (40), as well as an elevated risk of stroke recurrence. Patients in the highest TyG index group exhibited the greatest mortality risk (41). Furthermore, among patients with cerebral infarction in intensive care units, the TyG index demonstrated a linear relationship with mortality (42).

Our results also showed no significant association between the TyG index and sICH outcomes or the incidence of adverse events. This finding aligns with previous studies that assessed insulin resistance using the homeostasis model in patients receiving early IVT treatment for stroke (43). Similarly, prior research on triglycerides suggested no strong correlation between triglycerides and ICH in stroke patients who did not undergo thrombolysis (44). Based on these findings, we conclude that the TyG index is not a reliable predictor of hemorrhagic transformation in patients with cerebral infarction.

In this study, we observed statistically significant differences in age and APOA-1 levels among neurological outcome groups. Advanced age and lower APOA-1 levels were associated with poorer neurological recovery. Age is widely recognized as a critical factor in ischemic stroke, with elderly patients exhibiting higher mortality rates and worse functional recovery compared to younger patients (45, 46). Laboratory experiments using middle cerebral artery occlusion (MCAO) models in young and aged mice have explored the effects of age on stroke severity and underlying mechanisms. These studies revealed that aged mice experienced greater neutrophil-mediated blockages in the ischemic brain microcirculation, exacerbating perfusion deficits. Additionally, aged mice showed higher levels of atypical neutrophils in their blood, characterized by increased oxidative stress, phagocytosis, and prothrombotic properties (47). Furthermore, research has demonstrated that APOA-1, the primary component of high-density lipoprotein (HDL), facilitates cellular cholesterol efflux and reverse cholesterol transport, forming nascent HDL (48, 49). Higher HDL levels are associated with a reduced risk of stroke (50).

While most studies have investigated the relationship between the TyG index and the incidence of ischemic stroke, few have focused on its role in neurological recovery among stroke patients. Additionally, the cutoff values for the TyG index vary across studies (51–54). Based on findings from a Chinese study, we selected a cutoff value of 8.53, as the study identified this value as predictive of stroke prognosis in non-diabetic patients. Although the study did not distinguish between anterior and posterior circulation strokes, it is one of the few prospective studies to examine the correlation between the TyG index and stroke prognosis. After reviewing the literature, we adopted the 8.53 cutoff value. Future studies should aim to refine the TyG grouping criteria by conducting large-scale, multicenter investigations to identify the optimal specificity and sensitivity of this prognostic indicator.

Most current studies have not differentiated between subtypes of AIS, with some focusing only on the prognostic value of factors such as the TyG index in anterior circulation infarction patients. Posterior circulation infarction, however, carries a higher risk of disability and mortality compared to anterior circulation infarction and places a significant burden on families and society. Therefore, investigating prognostic indicators in posterior circulation thrombectomy patients is critically important (2, 5). Our study focuses specifically on patients who underwent thrombectomy, as successful thrombectomy rapidly restores cerebral blood flow, significantly improves neurological function in some patients, and enhances favorable outcomes. This information is also valuable for providing patients and their families with a clearer understanding of potential recovery trajectories after diagnosis. Consequently, this study exclusively included posterior circulation infarction patients who received interventional treatment, aiming to provide new evidence for prognostic indicators in Chinese patients with posterior circulation AIS treated with thrombectomy.

Posterior circulation infarction, as a critical subtype of ischemic stroke, presents complex prognostic outcomes but remains under-researched. Using real-world data, our study innovatively demonstrated the predictive value of the TyG index for neurological recovery three months after thrombectomy in posterior circulation infarction patients, addressing a significant research gap in this field. These findings establish a foundation for future multicenter validation studies and provide evidence for incorporating metabolic factors into stroke prediction models. Based on data from Chinese patients, our research offers specific evidence to optimize clinical practice and prediction models for Asian populations. However, further studies are required to generalize these findings to broader Asian and other racial populations. The TyG index, derived from simple laboratory measurements of lipid and glucose levels, is both practical and consistent. For patients with high TyG index values, closer monitoring and more aggressive therapeutic interventions may be warranted. However, whether maintaining the TyG index below 8.5 improves neurological outcomes and accelerates recovery remains uncertain and necessitates further investigation. For example, large-scale, multicenter, prospective randomized controlled clinical trials with stringent patient selection criteria are needed to minimize confounding factors and comprehensively analyze the predictive value of the TyG index.

Our study also has potential limitations, including selection bias and information bias. Additionally, despite clear inclusion and exclusion criteria, accurate measurement of key variables, and the use of appropriate statistical methods, the relatively small sample size may not fully represent the target population. Future studies with larger sample sizes and multicenter designs are required to validate the predictive value of the TyG index and its combined effects with other indicators. Furthermore, the TyG index is calculated from a single baseline blood test, which may largely reflect stress-induced hyperglycemia. Fasting blood glucose and triglyceride levels may also be influenced by pre-hospital interventions, potentially affecting the calculated TyG index to varying degrees. Finally, while this study adjusted for many potential confounding factors, the limited sample size may have resulted in unmeasured or inadequately adjusted confounders, which could influence the predictive value of the TyG index. Future studies with larger samples and more advanced regression algorithms are needed to further optimize these findings. Considering the significant role of the TyG index in predicting the 90 - day neurological recovery and mortality of these patients, future research could expand the sample size to multi - center studies, which would enhance the generalizability of our findings. Additionally, further exploration could focus on the underlying molecular mechanisms by which the TyG index influences post-stroke recovery, such as delving into its impact on the neurovascular unit and inflammatory responses. Long-term follow-up studies could also be conducted to determine the persistent effects of the TyG index on patients’ quality of life and recurrent stroke risk. These potential research directions aim to deepen our understanding of metabolic markers in stroke prognosis and guide more personalized post - stroke management.

In conclusion, this study supports that a higher TyG index is significantly associated with poor neurological outcome and death at 90 days in patients with acute posterior circulation ischemic stroke undergoing interventional therapy. For patients with acute posterior circulation cerebral infarction with high disability and high mortality, the data required by TyG index are simple and easy to obtain, which has potential value in helping clinicians to predict the recovery of neurological function of patients in the early stage. In the future, it is necessary to further expand the number of patients enrolled and more prospective experimental design, so as to make the TyG index more universal.

## Data Availability

The raw data supporting the conclusions of this article will be made available by the authors, without undue reservation.

## Glossary

ACS: Acute coronary syndrome
AIS: Acute ischemic stroke
APOA-1: Apolipoprotein A-1
APOB: Fasting apolipoprotein B
BAO: Basilar artery occlusion
BATMAN: The Basilar Artery Treatment and Management
CNSR II: The analysis of data from China National Stroke Registry II
CT: Computed tomography
CVD: Cardiovascular diseases
HDL: High-density lipoprotein
HEC: Hyperinsulinemic euglycemic clamp
HOMA: The homeostasis model assessment
IL-1β: Interleukin-1 beta
IQR: Interquartile range
IVGTT: the minimal model of the intravenous glucose tolerance test
IVT: Intravenous thrombolysis
MAPK: Mitogen-activated protein kinase
MCAO: Middle cerebral artery occlusion
MRI: Magnetic resonance imaging
mRS: modified Rankin Scale
NIHSS: National Institutes of Health Stroke Scale
PC-ASPECTS: Posterior circulation Alberta Stroke Program Early CT score
QQ plots: Quantile-Quantile Plots
QUICKI: The quantitative insulin sensitivity check index
sICH: Symptomatic intracranial hemorrhage
TNF-*α*: Tumor necrosis factor-alpha
TRLs: Triglyceride-rich lipoproteins
TyG: Triglyceride-glucose
VBAO: Vertebasilar artery occlusion
