# Correction: The predictive value of TyG index in patients with vertebrobasilar system thrombectomy

**DOI:** 10.3389/fneur.2025.1704033

**Published:** 2025-09-23

**Authors:** Jiazhen Wang, Honggang Ma, Xuanfei Jiang, Ru Pan, Bing Zhang, Ying Liu

**Affiliations:** ^1^Department of Neurology, Huzhou Central Hospital, The Fifth School of Clinical Medicine of Zhejiang Chinese Medical University, Huzhou, China; ^2^Department of Pathology, Huzhou Central Hospital, The Fifth School of Clinical Medicine of Zhejiang Chinese Medical University, Huzhou, China

**Keywords:** triglyceride-glucose index, vertebrobasilar system thrombectomy, modified Rankin score, ischemic stroke, metabolic-nutritional status

An incorrect **Funding** statement was provided. In the original funding statement, the following grants were erroneously included: “the Zhejiang Provincial Natural Science Foundation of China under Grant No. LQN25H090017 and the Huzhou Municipal Science and Technology Bureau Project under Grant No. 2024GYB04 from Ying Liu.”

The corrected funding statement appears below:

“The author(s) declare that financial support was received for the research and/or publication of this article. This research was supported by Zhejiang Provincial Medical and Healthcare Science and Technology Plan under Grant No. 2024KY1641 from Bing Zhang.”

The original version of this article has been updated.

