# Supplementary material for: The predictive value of TyG index in patients with vertebrobasilar system thrombectomy
Source: Front Neurol. 2025 Aug 15;16:1597323. doi: 10.3389/fneur.2025.1597323 (PMC12395455; doi:10.3389/fneur.2025.1597323)
Supplement: Supplementary file 1 [file Table_1.DOCX]

**Supplementary Material:**

## Table 1:Patient demographics and baseline characteristics

| **Characteristic** | **Triglyceride-glucose index** | | **p-value** |
| --- | --- | --- | --- |
|  | **＜8.53, N = 30^1^** | **≥8.53, N = 30^1^** |  |
| **Age** | 72 ± 9 | 67 ± 13 | 0.086^2^ |
| **sex** |  |  | 0.152^3^ |
| female | 6 (20.0%) | 11 (36.7%) |  |
| male | 24 (80.0%) | 19 (63.3%) |  |
| **Smoking** |  |  | 0.787^3^ |
| No | 20 (66.7%) | 19 (63.3%) |  |
| Yes | 10 (33.3%) | 11 (36.7%) |  |
| **Drinking** |  |  | 0.243^3^ |
| No | 24 (80.0%) | 20 (66.7%) |  |
| Yes | 6 (20.0%) | 10 (33.3%) |  |
| **History of hypertension** |  |  | 0.488^3^ |
| No | 4 (13.3%) | 6 (20.0%) |  |
| Yes | 26 (86.7%) | 24 (80.0%) |  |
| **History of atrial fibrillation** |  |  | 0.347^3^ |
| No | 22 (73.3%) | 25 (83.3%) |  |
| Yes | 8 (26.7%) | 5 (16.7%) |  |
| **History of diabetes mellitus** |  |  | 0.176^3^ |
| No | 22 (73.3%) | 17 (56.7%) |  |
| Yes | 8 (26.7%) | 13 (43.3%) |  |
| **History of ischemic stroke** |  |  | 0.424^4^ |
| No | 25 (83.3%) | 28 (93.3%) |  |
| Yes | 5 (16.7%) | 2 (6.7%) |  |
| **History of anticoagulants** |  |  | 0.353^4^ |
| No | 26 (86.7%) | 29 (96.7%) |  |
| Yes | 4 (13.3%) | 1 (3.3%) |  |
| **Lipoprotein a** | 20 ± 13 | 20 ± 18 | 0.839^2^ |
| **Apolipoprotein A-1** | 1.20 ± 0.20 | 1.18 ± 0.15 | 0.661^2^ |
| **Apolipoprotein B** | 0.72 ± 0.21 | 0.86 ± 0.23 | 0.018^2^ |
| **APOA-1/APOB** | 1.78 ± 0.56 | 1.45 ± 0.40 | 0.012^2^ |
| **Low density lipoprotein** | 76 ± 33 | 88 ± 31 | 0.159^2^ |
| **Triglycerides** | 0.76 ± 0.22 | 1.82 ± 0.89 | <0.001^2^ |
| **Fasting glucose** | 6.1 ± 1.6 | 8.3 ± 3.5 | 0.003^2^ |
| **Cause of stroke** |  |  | 0.624^4^ |
| Atherosclerosis | 14 (46.7%) | 16 (53.3%) |  |
| Cardiac embolism | 11 (36.7%) | 9 (30.0%) |  |
| Other causes | 2 (6.7%) | 4 (13.3%) |  |
| Unknown | 3 (10.0%) | 1 (3.3%) |  |
| **Time from symptom onset to groin puncture** | 368 ± 281 | 360 ± 206 | 0.900^2^ |
| **Time from symptom onset to recanalization** | 433 ± 284 | 432 ± 219 | 0.992^2^ |
| **Puncture to recanalization time** | 65 ± 30 | 72 ± 47 | 0.478^2^ |
| **Door to recanalization time** | 237 ± 170 | 203 ± 81 | 0.331^2^ |
| **First thrombectomy attempt** |  |  | 0.822^3^ |
| aspiration | 6 (20.0%) | 7 (23.3%) |  |
| stenting | 16 (53.3%) | 17 (56.7%) |  |
| stenting+stenting | 8 (26.7%) | 6 (20.0%) |  |
| **Location of intracranial artery occlusion** |  |  | 0.794^4^ |
| distal | 1 (3.3%) | 2 (6.7%) |  |
| middle | 4 (13.3%) | 5 (16.7%) |  |
| tip of the basilar artery occlusion | 25 (83.3%) | 23 (76.7%) |  |
| **Number of thrombectomy maneuvers** |  |  | >0.999^3^ |
| 1 | 17 (56.7%) | 17 (56.7%) |  |
| 2 | 13 (43.3%) | 13 (43.3%) |  |
| **Intravenous thrombolysis** |  |  | 0.605^3^ |
| No | 15 (50.0%) | 17 (56.7%) |  |
| Yes | 15 (50.0%) | 13 (43.3%) |  |
| **Tirofiban** |  |  | 0.405^3^ |
| No | 22 (73.3%) | 19 (63.3%) |  |
| Yes | 8 (26.7%) | 11 (36.7%) |  |
| **BETMAN score** |  |  | >0.999^3^ |
| 0-8 | 21 (70.0%) | 21 (70.0%) |  |
| 9-10 | 9 (30.0%) | 9 (30.0%) |  |
| **ASPECT** |  |  | 0.067^3^ |
| 0-8 | 4 (13.3%) | 10 (33.3%) |  |
| 9-10 | 26 (86.7%) | 20 (66.7%) |  |
| **Baseline NIHSS score** | 31.5 ± 8.5 | 32.5 ± 6.7 | 0.625^2^ |
| **MRS** |  |  | 0.017^3^ |
| 0-2 | 16 (53.3%) | 7 (23.3%) |  |
| 3-6 | 14 (46.7%) | 23 (76.7%) |  |
| ^1^Mean ± SD; n (%) | | | |
| ^2^Welch Two Sample t-test | | | |
| ^3^Pearson's Chi-squared test | | | |
| ^4^Fisher's exact test | | | |
